# mHealth innovations as health system strengthening tools: 12 common applications and a visual framework

**DOI:** 10.9745/GHSP-D-13-00031

**Published:** 2013-08-06

**Authors:** Alain B Labrique, Lavanya Vasudevan, Erica Kochi, Robert Fabricant, Garrett Mehl

**Affiliations:** a Johns Hopkins Bloomberg School of Public Health, Baltimore, MD, USA; b United Nations Children's Fund (UNICEF), New York City, NY, USA; c frog Design, New York City, NY, USA; d World Health Organization, Geneva, Switzerland

## Abstract

This new framework lays out 12 common mHealth applications used as health systems strengthening innovations across the reproductive health continuum.

The rapid proliferation of mHealth projects—albeit mainly pilot efforts—has generated considerable enthusiasm among governments, donors, and implementers of health programs.^1^ In many instances, these pilot projects have demonstrated conceptually how mHealth can alleviate specific health system constraints that hinder effective coverage of health interventions.

Large-scale implementation or integration of these mHealth innovations into health programs has been limited, however, by a shortage of empirical evidence supporting their value in terms of cost, performance, and health outcomes.^1^-^4^ Governments in low- and middle-income countries face numerous challenges and competing priorities, impeding their ability to adopt innovations.^2^ Thus, they need robust, credible evidence about mHealth projects in order to consider mHealth alongside essential health interventions, and guidance about which mHealth solutions they should consider to achieve broader health system goals.^2^ Their tolerance for system instability or failure can be low, even when the status quo may be equally, or more, unreliable.

Current larger-scale effectiveness and implementation research initiatives are working to address the evidence gaps and to demonstrate the impact of mHealth investments on health system targets.^1^ Other efforts are underway to synthesize such findings.^5^

## MHEALTH AS A HEALTH SYSTEMS STRENGTHENING TOOL

Recent mHealth reviews have proposed that innovators focus on the public health principles underlying mHealth initiatives, rather than on specific mHealth technologies.^6^ International agencies and research organizations have also endeavored to frame mHealth interventions within the broader context of health system goals or health outcomes.^2^ The term “health system” includes all activities in which the primary purpose is to promote, restore, or maintain health.^7^ Some elements of a framework for evaluating health systems performance by relating the goals of the health system to its essential functions have been proposed previously, which we believe can serve as a model for articulating and justifying mHealth initiatives and investments.^7^

Applying a health systems lens to the evaluation of mHealth initiatives requires different indicators and methodologies, shifting the assessment from whether the mHealth initiative “works” to process evaluation or proxy indicators of the health outcome(s) of interest. This new way of thinking would facilitate selection of mHealth tools that are appropriate for identified challenges. In other words, it would drive people to first identify the key obstacles, or constraints, to delivering proven health interventions effectively, and to then apply appropriate mHealth strategies that could overcome these health system constraints.^8^

Presenting mHealth as a range of tools for overcoming known health system constraints, as a health systems “catalyst,” may also improve communication between mHealth innovators and health program implementers. Communicating mHealth technologies as tools that can enhance delivery of life-saving interventions through improvements in health systems performance, such as coverage, quality, equity, or efficiency, will resonate with health decision-makers.^7^

Hence, rather than being perceived as siloed, stand-alone solutions, mHealth strategies should be viewed as integrable systems that should fit into existing health system functions and complement the health system goals of: health service provision; a well-performing health workforce; a functioning health information system; cost-effective use of medical products, vaccines, and technologies; and accountability and governance.^9^

mHealth should be integrated into existing health system functions, rather than as stand-alone solutions.

## A SHARED FRAMEWORK TO EXPLAIN MHEALTH INNOVATIONS

The absence of a shared language and approach to describe mHealth interventions will continue to hinder efforts to identify, catalog, and synthesize evidence across this complex landscape. The lack of a common framework also makes it hard to explain mHealth innovations to mainstream health-sector stakeholders.

mHealth researchers and implementers at the World Health Organization (WHO), the Johns Hopkins University Global mHealth Initiative, the United Nations Children's Fund (UNICEF), and frog Design have jointly developed the “mHealth and ICT Framework” to describe mHealth innovations in the reproductive, maternal, newborn, and child health (RMNCH) field, in which mobile health technologies have been broadly implemented over the last decade across the developing world.

The framework builds on prior efforts to describe types and uses of mHealth generally, such as in the WHO global survey on eHealth^2^ and in the mHealth Alliance's typology for mHealth services in the maternal and newborn health field.^10^ These previous efforts, however, have focused more explicitly on the type of actor (client, provider, or health system) and location of the mHealth activity (community, facility, or health information system). Some of these descriptions provide details about the use of specific mobile functions (such as toll-free help lines) to accomplish particular health goals, although other functions could have been used to accomplish the same goals and, over time, the functions described could be superseded by newer technologies. Furthermore, their classification approaches have not provided stakeholders with the tools to enable them to understand the diverse ways in which specific mobile functions could be employed for a particular health purpose.

**Figure 1.**
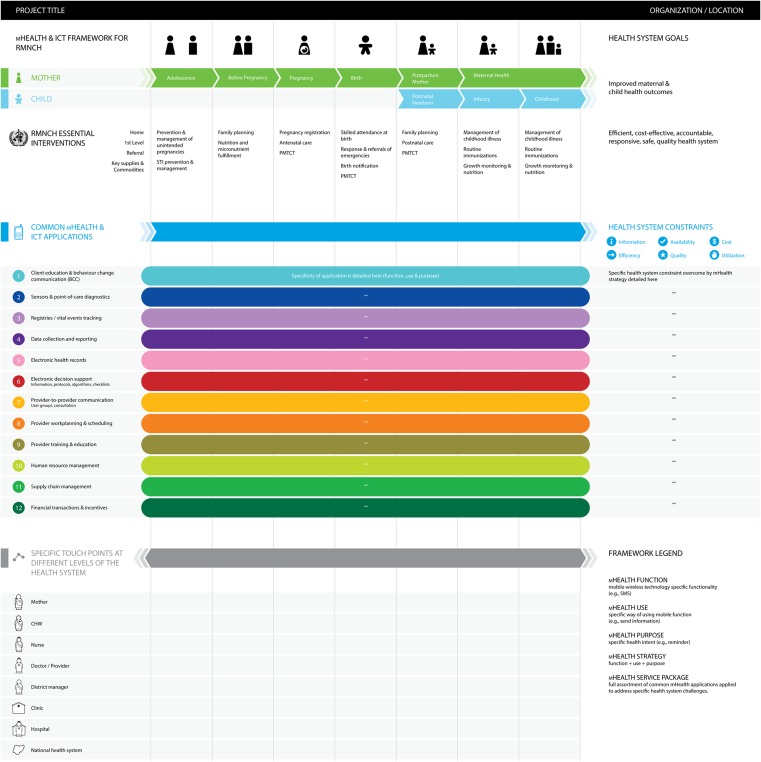
The mHealth and ICT Framework for RMNCH Abbreviations: CHW, community health worker; ICT, information and communications technology; PMTCT, prevention of mother-to-child transmission of HIV; RMNCH, reproductive, maternal, newborn, and child health.

Our framework is constructed around standard health system goals and places intended users and beneficiaries in central focus, against the context of the proposed mHealth service package (Figure 1). By describing a specific mHealth strategy or approach, the framework visually depicts the when, for whom, what is being done to alleviate which constraints, and the how of the strategy. The framework comprises 2 key components:

1. A place to depict the specifics of the mHealth intervention, described as one or more common mHealth or information and communications technology (ICT) applications used to target specific health system challenges or constraints within specific areas of the RMNCH continuum of care (Figure 1, upper section).
2. A visual depiction of mHealth implementation through the concept of “touch points,” or points of contact, which describe the specific mHealth interactions across health system actors (for example, clients, providers), locations (such as clinics or hospitals), and timings of interactions and data exchange (Figure 1, lower section).

## 12 COMMON MHEALTH AND ICT APPLICATIONS

The first part of the framework aims to address a previously identified challenge in mHealth: to systematically describe the constituent parts of an mHealth strategy or platform.^11^ To do this, we define relationships between common applications of mHealth and ICT and the health systems constraints that they address.^2^,^12^-^13^

**Figure 2.**
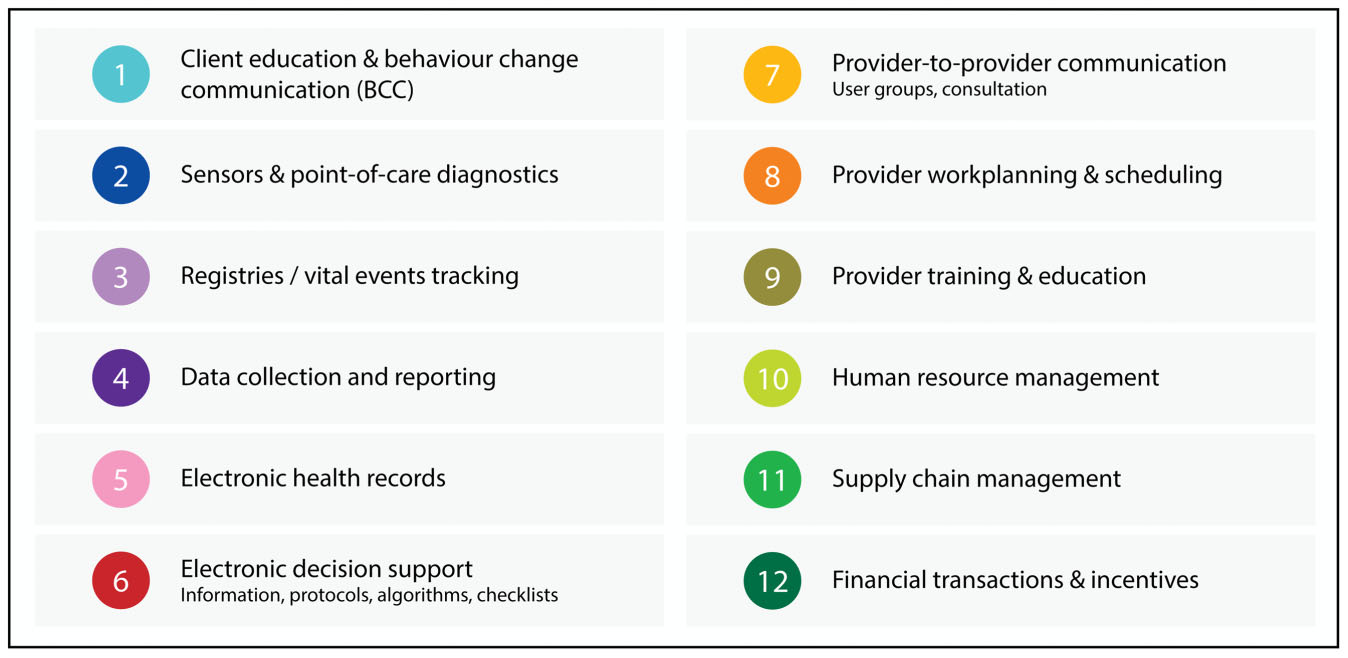
Twelve Common mHealth and ICT Applications

Our list of 12 common mHealth applications has been vetted, through multiple iterations, by a wide group of mHealth stakeholders and thought leaders, ranging from academic researchers to program and policy implementers. Although a few mHealth projects deploy a single application, most comprise a package of 2 or more applications (Figure 2). In addition, mHealth projects employ 1 or more mobile phone functions—such as short message service (SMS), interactive voice response (IVR)—to accomplish the common applications (Table 1).

**Table 1.** Examples of Mobile Phone Functions Used in Common mHealth and ICT Applications

	Common mHealth and ICT Applications	Examples of Mobile Phone Functions
1	Client education and behavior change communication (BCC)	• Short Message Service (SMS)
		• Multimedia Messaging Service (MMS)
		• Interactive Voice Response (IVR)
		• Voice communication/Audio clips
		• Video clips
		• Images
2	Sensors and point-of-care diagnostics	• Mobile phone camera
		• Tethered accessory sensors, devices
		• Built-in accelerometer
3	Registries and vital events tracking	• Short Message Service (SMS)
		• Voice communication
		• Digital forms
4	Data collection and reporting	• Short Message Service (SMS)
		• Digital forms
		• Voice communication
5	Electronic health records	• Digital forms
		• Mobile web (WAP/GPRS)
6	Electronic decision support (information, protocols, algorithms, checklists)	• Mobile web (WAP/GPRS)
		• Stored information “apps”
		• Interactive Voice Response (IVR)
7	Provider-to-provider communication (user groups, consultation)	• Short Message Service (SMS)
		• Multimedia Messaging Service (MMS)
		• Mobile phone camera
8	Provider work planning and scheduling	• Interactive electronic client lists
		• Short Message Service (SMS) alerts
		• Mobile phone calendar
9	Provider training and education	• Short Message Service (SMS)
		• Multimedia Messaging Service (MMS)
		• Interactive Voice Response (IVR)
		• Voice communication
		• Audio or video clips, images
10	Human resource management	• Web-based performance dashboards
		• Global Positioning Service (GPS)
		• Voice communication
		• Short Message Service (SMS)
11	Supply chain management	• Web-based supply dashboards
		• Global Positioning Service (GPS)
		• Digital forms
		• Short Message Service (SMS)
12	Financial transactions and incentives	• Mobile money transfers and banking services
		• Transfer of airtime minutes

Abbreviations: GPRS, General Packet Radio Service; WAP, Wireless Application Protocol.

### 1. Client Education and Behavior Change Communication

This series of mHealth strategies focuses largely on the client, offering a novel channel to deliver content intended to improve people's knowledge, modify their attitudes, and change their behavior. Targeted, timely health education and actionable health information—delivered through SMS, IVR, audio, and/or videos that engage 1 or more actors (such as a pregnant woman, a husband, family, community)—influences health behaviors, such as adherence to medication or use of health services.^3^^,^^14^ The Mobile Alliance for Maternal Action (MAMA) is an example of an mHealth service package that provides gestational age-appropriate health information to pregnant women and new mothers on their family's mobile phone.^15^

Most mHealth interventions in this category capitalize on people's ubiquitous access to mobile phones to increase their exposure to, and reinforce, health messages. In some instances, these types of interventions also enable clients to seek more information based on their interest in a particular message—for example, through a higher level of engagement with a call-center counselor.^4^

Other mHealth interventions use mobile functions such as voice, video or audio clips, and images to enhance the effectiveness of in-person counseling, which is of particular value among low-literacy populations. Such examples include the BBC World Trust Mobile Kunji project^16^ and Dimagi's CommCare Health Worker systems.^17^-^18^

### 2. Sensors and Point-of-Care Diagnostics

Harnessing the inherent computing power of mobile phones or linking mobile phones to a connected, but independent, external device can facilitate remote monitoring of clients, extending the reach of health facilities into the community and into clients' homes. Novel sensors and technologies are being developed to conduct, store, transmit, and evaluate diagnostic tests through mobile phones, from relatively simple tests, such as blood glucose measurements for diabetes management, to sophisticated assays, such as electrocardiograms (ECGs), in situations where the patient and provider are far removed from one another. These technologies also can store frequent longitudinal measures for later review during a patient-provider visit and monitor a patient's vital signs continuously and automatically, triggering a response when the device detects anomalous signals. Examples of such mHealth initiatives include the “ubiquitous health care” service in South Korea^19^ that uses sensor technology to monitor patient health remotely and AliveCor,^20^ a clinical grade, 2-lead ECG running on a mobile phone, recently approved by the U.S. Food and Drug Administration (FDA), that allows physicians to view and assess cardiac health at the point-of-care. These kinds of interventions are increasingly common in high-income settings but are less common in resource-limited contexts.

New tests are being developed and evaluated to allow diagnostics to be conducted through mobile phones, from simple blood glucose tests to sophisticated electrocardiograms.

### 3. Registries and Vital Events Tracking

Mobile phone-based registration systems facilitate the identification and enumeration of eligible clients for specific services, not only to increase accountability of programs for providing complete and timely care but also to understand and overcome disparities in health outcomes.^21^ These are most often used for registering pregnancy and birth but also can be used for tracking individuals with specific health conditions, by age groups or other characteristics. Tracking vital events (births and deaths) supports the maintenance of population registries and determination of key development indicators, such as maternal and neonatal mortality. Such mobile registries issue and track unique identifiers and common indicators, link to electronic medical records, and enable longitudinal population information systems and health reporting.

One such registry is the Mother and Child Tracking System (MCTS) in India^22^ that registers pregnant women, using customized mobile phone-based applications, to help strengthen accountability for eligible clients to receive all scheduled health services (for example, 3–4 antenatal checkups, postnatal visits, and childhood vaccinations); both frontline health workers and their clients receive SMS reminders about scheduled services. Another example is UNICEF's birth registration system in Uganda, which uses RapidSMS to maintain a central electronic database of new births, updated using information transmitted via SMS, to overcome obstacles with the previously inefficient paper-based system.^23^-^24^

### 4. Data Collection and Reporting

Among the earliest global mHealth projects were those that allowed frontline workers and health systems to move from paper-based systems of ledgers, rosters, and aggregated reports to the near-instantaneous reporting of survey or patient data. Aggregation of information can occur at the server to analyze health system or disease statistics, by time, geographic area, or worker. In addition to optimizing the primary research or program monitoring and evaluation efforts of researchers, these types of mHealth initiatives reduce the turnaround time for reporting district-, local-, state-, or national-level data, which is useful for supervisors and policy makers. Countries such as Bangladesh, Rwanda, and Uganda are developing and enforcing national health information technology policies to improve the standardization and interoperability of public health data collection systems across government agencies and nongovernmental organizations (NGOs).

Among the earliest mHealth projects were those that allowed collection of survey or patient data through mobile phones.

Platforms commonly used to develop data collection systems include Open Data Kit (ODK) and FrontlineSMS.^25^-^26^ The Formhub platform makes it easy for developers to use Microsoft Excel to create electronic forms, which can be deployed via Web forms or Android phones, with sophisticated server-side facilities for data aggregation, sharing, and visualization.^27^ A large number of commercial systems exist for the range of mobile operating systems (iOS, Android, HTML5), and they often present user-friendly interfaces, such as Magpi,^28^ that allow people to easily design mobile questionnaires. In Formhub and Magpi, forms can be shared with mobile data collectors and the data visualized in real time on a map, as the data are collected.

National-level systems have also been developed for widespread use, such as the open-source District Health Information Software 2 (DHIS2) system, currently used in a number of countries for routine health collection and reporting.^29^ In addition to being integrated into national health information systems, DHIS2 accepts data from authorized mobile devices and can allow management of data at the individual (such as district) or aggregate (national) levels.^29^

### 5. Electronic Health Records

Electronic health records (EHRs) used to be connected only to the facilities they served, allowing clinical staff to access patient records through fixed desktop computers. But the advent of mHealth has redefined the boundaries of the EHR; now, health workers can electronically register the services they provide and submit point-of-care test results through mHealth systems to update patient histories from the field. Rural health workers at the point-of-care (for example, in rural clinics or in the patient's home) can access and contribute to longitudinal health records, allowing continuity of care that was previously impossible in non-hospital-based settings.^30^ Server-side algorithms to identify care gaps or trends in key indicators, such as weight loss or blood-glucose fluctuations, shift the onerous burden of identifying patterns and generating cues-to-action away from human reviewers.

OpenMRS, a popular mHealth-enhanced EHR, allows frontline health workers to access information from a patient's health record using a mobile device and to contribute information into the health record—for example, about field-based tuberculosis (TB) treatment.^30^ Other systems, such as RapidSMS or ChildCount+, might not be linked to a clinical file but still can maintain longitudinal client histories, such as antenatal care documentation, infant and child growth records, and digital vaccine records.^23^,^31^-^32^

### 6. Electronic Decision Support: Information, Protocols, Algorithms, Checklists

Ensuring providers' adherence to protocols is a paramount challenge to implementing complex care guidelines. In particular, shifting tasks, such as screening responsibilities, from clinicians to frontline health workers often entails adapting procedures designed for clinical workers to cadres with limited formal training. mHealth initiatives that incorporate point-of-care decision support tools with automated algorithm- or rule-based instructions help ensure quality of care in these task-shifting scenarios by prompting frontline health workers to follow defined guidelines.

Point-of-care decision support tools through mobile phones can help ensure quality of care.

Electronic decision support tools also can be used to identify and prioritize high-risk clients for health care, targeting interventions in resource-limited contexts. e-IMCI (electronic-Integrated Management of Childhood Illnesses), for example, provides community health workers with mobile phone-based, step-by-step support to triage and treat children according to WHO protocols for the diagnosis and treatment of common childhood diseases.^33^-^34^ In addition, several groups are developing mobile phone-based checklists to help reduce clinical errors or to ensure quality of care at the point of service delivery.^35^

### 7. Provider-to-Provider Communication: User Groups, Consultation

Voice communication—one of the simplest technical functions of mobile phones—is among the most transformative applications in an mHealth service package, allowing providers to communicate with one another or across hierarchies of technical expertise. Once a key feature of telemedicine strategies, provider-to-provider communication by mobile phone can be used to coordinate care and provide expert assistance to health staff, when and where it is needed. Furthermore, communication is not limited to voice only; mobile phones allow the exchange of images or even sounds (for example, through digital auscultation, extending the reach of the traditional stethoscope) for immediate remote consultation.

Providers can use simple voice communication through mobile phones to coordinate care and provide expert assistance.

Current examples of provider-to-provider communication include the establishment of “Closed User Group” networks in Ghana, Liberia, and Tanzania by the NGO Switchboard, by which members of each mobile phone group can communicate with one another at heavily discounted rates, or for free.^36^-^37^ In Nigeria, an mHealth feedback loop between rural clinics and diagnostic laboratories reduces the turnaround time between HIV testing and result reporting to facilitate prompt care and referral.^38^

### 8. Provider Work Planning and Scheduling

Work planning and scheduling tools help keep health care workers informed through active reminders of upcoming or due/overdue services, and they promote accountability by prioritizing provider follow-up. In low-resource settings, there often is a shortage of providers, making it a challenge to provide systematic population follow-up using traditional paper-based methods. mHealth systems can facilitate the scheduling of individuals listed in population registries (described in application number 3) for household-based outreach visits.

Examples of this application include scheduling antenatal and postnatal care visits; alerting providers or supervisors about missed vaccinations or reduced adherence to medication regimens; and following up about medical procedures, such as circumcision or long-acting and permanent family planning methods. Provider work planning tools are common in many mHealth service packages, such as the scheduling functions of TxtAlert^39^ and the MoTech “Mobile Midwife Service” that alerts nurses about clients who are due or overdue for care, to prevent missed appointments and delays in service provision.^40^

### 9. Provider Training and Education

Continuing medical education has been a mainstay of quality of care in high-income settings. Now, mobile devices are being used to provide continued training support to frontline and remote providers, through access to educational videos, informational messages, and interactive exercises that reinforce skills provided during in-person training. They also allow for continued clinical education and skills monitoring—for example, through quizzes and case-based learning.

Applications for provider training include eMOCHA,^41^-^42^ a platform that allows frontline health workers in rural Uganda to select streaming video content as part of continuing education. eMOCHA recently released “TB Detect,” a free application for Android devices in the Google Play Store, allowing providers to access continually updated educational content about tuberculosis prevention, detection, and care.

### 10. Human Resource Management

Community health workers often work among rural populations, with only sporadic contact with supervisory staff. Web-based dashboards allow supervisors to track the performance of community health workers individually or at the district/regional/national level, either by noting the volume of digital productivity or by real-time GPS tracking of workers as they perform their field activities. This enables supportive supervision to those workers who may be lagging in their performance, while also enabling the recognition and reward of exceptional field staff.

These approaches are embedded within a number of mHealth service packages, such as Rwanda's mUbuzima, which helps supervisors monitor community health worker performance and provide performance-based incentives,^43^-^44^ and UNICEF's RapidSMS in Rwanda, which enables supervisors to monitor exchange of SMS messages between community health workers and a central server, thereby measuring service accountability and responsiveness of community health workers.^24^^,^^45^

### 11. Supply Chain Management

mHealth tools to track and manage stocks and supplies of essential commodities have received significant global attention. Relatively simple technologies that allow remote clinics or pharmacies to report daily stock levels of drugs and supplies, or to request additional materials electronically, have been implemented in a number of countries.

Many countries use mHealth tools to track and manage stocks of health commodities.

In Tanzania, at least 130 clinics are using the SMS for Life mHealth supply chain system to prevent stockouts of essential malaria drugs.^46^-^48^ Pharmacists and other service providers are trained to send their district-level supervisors a structured text message at the end of each week to report stock levels of key commodities including anti-malarials. The supervisors can then take necessary actions to redistribute supplies, circumventing a potential crisis.

In addition, a number of projects have developed mHealth strategies to reduce the risk of purchasing counterfeit drugs in countries where this is a major public health threat.^49^ Companies such as Sproxil have partnered with drug manufacturers to provide mHealth authentication services to the purchasing public.^49^ These strategies may help improve supply chain transparency and bolster a system's ability to be proactive and responsive to supply needs, with district or national-level visibility of performance.

### 12. Financial Transactions and Incentives

mHealth and mFinance are converging rapidly in the domain of financial transactions to pay for health care, supplies, or drugs, or to make demand- or supply-side incentive schemes easier to deploy and scale. These strategies focus on decreasing financial barriers to care for clients, and they are testing novel ways of motivating providers to adhere to guidelines and/or provide higher quality care. Mobile financial transactions are becoming increasingly common. For example, a single African network operator, MTN, estimated having 7.3 million mobile money clients in mid-2012.^50^ Thus, providing incentives to clients to use particular areas of health services will be increasingly attractive (for example, for institutional deliveries or vaccines, vouchers to subsidize health services, universal health insurance schemes, and mobile banking for access to resources for health services^51^). Mobile-based cash vouchers have also been used where mobile money is not standard, as illustrated by the use of conditional cash transfers in Pakistan to provide families with an incentive to immunize their infants.^52^-^53^

## PLACING THE 12 APPLICATIONS WITHIN THE RMNCH FRAMEWORK

One illustration of the application of component parts of our framework is the display of mHealth projects working within the RMNCH continuum to improve health systems functions. Specifically, the common mHealth applications capture the core uses of mobile technology and their contribution toward meeting health system needs. Health system challenges and constraints in the framework embrace and draw from concepts articulated in the WHO building blocks of health systems (service delivery, health workforce, health information systems, access to essential medicines, financing, and leadership/governance).^54^ The framework's intended audience ranges from mHealth projects—to help locate their work within a broader context of mHealth in the RMNCH landscape—to stakeholders in government, implementation, or donor communities.

In brief, the framework begins with the RMNCH continuum of care for women of reproductive age and their children to establish “when” during the reproductive life cycle the mHealth project will focus.^55^ In other words, it identifies the beneficiary targets of the mHealth strategy, such as adolescents or pregnant women, as well as the intended users of the system, such as community health workers or district supervisors.

Next, the framework identifies which RMNCH essential interventions (including preventive and curative care for improved maternal and child health outcomes) the mHealth approach will target, such as pregnancy registration or management of childhood illnesses.^56^-^57^ This helps maintain focus on the needs of the health system and on the intervention that the mHealth approach is facilitating,^7^ rather than on the technology being used.

Rather than focus on technology, our new mHealth framework places emphasis on addressing health system needs.

**Figure 3.**
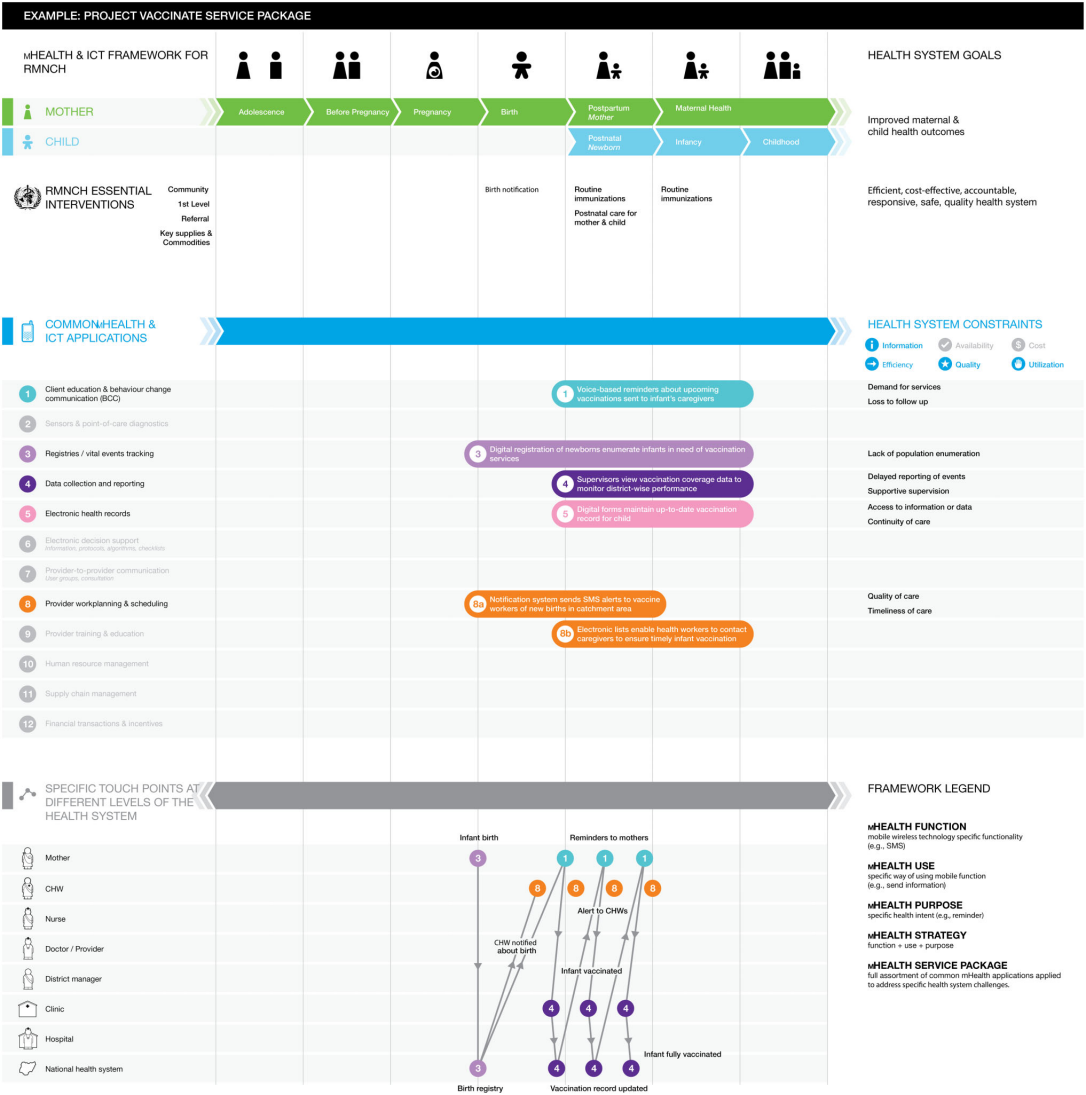
Sample Application of the mHealth and ICT Framework for RMNCH Abbreviations: CHW, community health worker; ICT, information and communications technology; RMNCH, reproductive, maternal, newborn, and child health. The fictional “Project Vaccinate” is an mHealth system that integrates 5 of the 12 common mHealth applications to identify newborns and support families and community health workers in ensuring timely and complete vaccination.

The common mHealth and ICT applications used by the project are indicated by horizontal, colored bars running across the RMNCH continuum of care, from adolescence to pregnancy and birth to childhood. The framework also incorporates space (to the right of the colored bars) to succinctly describe the specific health system constraints that the project is addressing (for example, “delayed reporting of events”). The framework includes categories of common health system challenges, such as information, availability, and cost. Finally, the “touch points” layer in the lower portion of the framework allows for mapping the mHealth-facilitated interactions among health system actors (for example, client, provider, manager, hospital, national health system).^58^ See Figure 3 for an illustrative example of the fictional “Project Vaccinate.”

A detailed description of the components and use of the framework are beyond the scope of this commentary. In the near future, we will provide an updated framework and user guide as web-based, online tools that mHealth innovators and other stakeholders can use. Thus, the framework would serve to map and catalog mHealth service packages used across the RMNCH continuum, describing their work using a common language. As mHealth stakeholders begin to use this tool and employ this common language to describe their mHealth innovations, we expect to foster improved understanding between mHealth innovators and mainstream health system program and policy planners.

This framework not only helps individual projects articulate their mHealth strategies through a shared tool but also facilitates identification of gaps in innovation, solutions, and implementation activity by overlaying multiple projects onto a single visualization. Any remaining blank spaces in the central area of the framework will signal areas of the continuum where future mHealth attention and investment may be warranted. This would also help identify common mHealth applications not yet utilized to target particular health system constraints.

The new mHealth framework will help identify gaps in mHealth innovation.

Ultimately, we hope these initial efforts at building consensus around a common taxonomy and framework will help overcome misgivings that mHealth innovations are the new “verticals” of this decade. Innovations in this space should be viewed not as independent, disconnected strategies but as vehicles to overcome persistent health system constraints. mHealth applications in this framework largely serve to catalyze the effective coverage of proven health interventions.

Although shared frameworks are critical to communicating value, continued efforts to evaluate and generate evidence of mHealth impact are also necessary to sustain growth and mainstreaming of these solutions. These efforts should be complementary to improving the quality of deployments through end-user engagement, stakeholder inclusion, and designing for scale.^59^
